# Seroprevalence Surveys for Anti-SARS-CoV-2 Antibody in Different Populations in Taiwan With Low Incidence of COVID-19 in 2020 and Severe Outbreaks of SARS in 2003

**DOI:** 10.3389/fimmu.2021.626609

**Published:** 2021-05-18

**Authors:** Wen-Pin Tseng, Jhong-Lin Wu, Chen-Chi Wu, Kuan-Ting Kuo, Chien-Hao Lin, Ming-Yi Chung, Ya-Fan Lee, Bey-Jing Yang, Chien-Hua Huang, Shey-Ying Chen, Chong-Jen Yu, Shyr-Chyr Chen, Po-Ren Hsueh

**Affiliations:** ^1^ Department of Emergency Medicine, National Taiwan University Hospital, National Taiwan University College of Medicine, Taipei, Taiwan; ^2^ Department of Otolaryngology, National Taiwan University Hospital, National Taiwan University College of Medicine, Taipei, Taiwan; ^3^ Department of Medical Research and Department of Integrated Surgery, Hsinchu Biomedical Science Park Medical Center (National Taiwan University Hospital Hsin-Chu Biomedical Park Branch), Hsinchu, Taiwan; ^4^ Department of Pathology, National Taiwan University Hospital, National Taiwan University College of Medicine, Taipei, Taiwan; ^5^ Department of Laboratory Medicine, National Taiwan University Hospital Hsin-Chu Biomedical Park Branch, Hsinchu, Taiwan; ^6^ Department of Laboratory Medicine, National Taiwan University Hospital, National Taiwan University College of Medicine, Taipei, Taiwan; ^7^ Center for Quality Management, National Taiwan University Hospital, College of Medicine, National Taiwan University, Taipei, Taiwan; ^8^ Department of Internal Medicine, National Taiwan University Hospital, National Taiwan University College of Medicine, Taipei, Taiwan; ^9^ Department of Internal Medicine, National Taiwan University Hospital Hsin-Chu Biomedical Park Branch, Hsinchu, Taiwan

**Keywords:** SARS-CoV-2, COVID-19, seroprevalence, cross-reactivity, SARS

## Abstract

Accurate detection of anti-SARS-CoV-2 antibodies provides a more accurate estimation of incident cases, epidemic dynamics, and risk of community transmission. We conducted a cross-sectional seroprevalence study specifically targeting different populations to examine the performance of pandemic control in Taiwan: symptomatic patients with epidemiological risk and negative qRT-PCR test (Group P), frontline healthcare workers (Group H), healthy adult citizens (Group C), and participants with prior virologically-confirmed severe acute respiratory syndrome (SARS) infection in 2003 (Group S). The presence of anti−SARS−CoV−2 total and IgG antibodies in all participants were determined by Roche Elecsys^®^ Anti−SARS−CoV−2 test and Abbott SARS-CoV-2 IgG assay, respectively. Sera that showed positive results by the two chemiluminescent immunoassays were further tested by three anti-SARS-CoV-2 lateral flow immunoassays and line immunoassay (MIKROGEN *recom*Line SARS-CoV-2 IgG). Between June 29 and July 25, 2020, sera of 2,115 participates, including 499 Group P participants, 464 Group H participants, 1,142 Group C participants, and 10 Group S participants, were tested. After excluding six false-positive samples, SARS-CoV-2 seroprevalence were 0.4, 0, and 0% in Groups P, H, and C, respectively. Cross-reactivity with SARS-CoV-2 antibodies was observed in 80.0% of recovered SARS participants. Our study showed that rigorous exclusion of false-positive testing results is imperative for an accurate estimate of seroprevalence in countries with previous SARS outbreak and low COVID-19 prevalence. The overall SARS-CoV-2 seroprevalence was extremely low among populations of different exposure risk of contracting SARS-CoV-2 in Taiwan, supporting the importance of integrated countermeasures in containing the spread of SARS-CoV-2 before effective COVID-19 vaccines available.

## Introduction

Coronavirus disease 2019 (COVID-19), which emerged at the end of 2019 in China and is caused by severe acute respiratory syndrome coronavirus 2 (SARS-CoV-2), has rapidly evolved to a pandemic and impacts healthcare, public health, and the socioeconomic system tremendously (1, 2). The risk of a community outbreak of COVID-19 in Taiwan is especially high because of its geographic proximity and frequent person-to-person contacts with China. By virtue of its experience in coping with the severe impact of severe acute respiratory syndrome (SARS) in 2003 (3, 4), Taiwan responded to this global public health emergency promptly and has maintained a record of limited community transmission of COVID-19 (5). The first confirmed COVID-19 case in Taiwan was imported from China on January 20, 2020, and was identified at the airport entry quarantine system (6). Though cases from sporadic family clusters and one nosocomial outbreak have been reported, most of the confirmed COVID-19 cases in Taiwan were imported from aboard. As of August 28, 2020, the latest confirmed indigenous COVID-19 case reported by Taiwan authorities was on April 13, 2020. Meanwhile, only 487 confirmed cases, including 55 indigenous cases, have been reported in Taiwan (7). The number of confirmed COVID-19 cases per million Taiwan population was 20.4, ranking 204 out of 209 countries (8).

Although early success in the control of the COVID-19 pandemic was achieved, Taiwan faces an increasing risk of COVID-19 community transmission due to the rapid spread of SARS-CoV-2 globally and the rising influx of business and returning travelers. Furthermore, the risk of circulating SARS-CoV-2 in the community from untested mild or asymptomatic patients or from symptomatic patients with false-negative results by real-time reverse transcriptase-polymerase chain reaction (qRT-PCR) assay remains a serious concern (9). All these issues could seriously confound the estimation of incident cases, epidemic dynamics, and ongoing risk of COVID-19 community transmission from current viral nucleic acid testing-based reporting data (10–14).

Serological testing, i.e., detection of anti-SARS-CoV-2 antibodies in a person’s blood, has been proposed as a useful laboratory tool in the diagnosis of current or recovered COVID-19 infection, screening of recovered COVID-19 patients for convalescent plasma therapy, SARS-CoV-2 seroprevalence studies, and monitoring immune responses to COVID-19 vaccine candidates (15). Although a false-positive result has been reported, the detection sensitivity of many serological tests for COVID-19 infection is high, especially after 2–3 weeks of symptom onset (16–19). Therefore, population-based serological tests might provide a more accurate estimation of SARS-CoV-2 transmission and disease burden that comprehensively include COVID-19 patients that are asymptomatic, with false negative qRT-PCR testing results, and with qRT-PCR-confirmed infection.

In the seroprevalence study, we conducted a serological survey targeting three groups of population with two automated immunoassays simultaneously: (i) symptomatic patients with risk of SARS-CoV-2 exposure, (ii) healthcare workers (HCWs) responsible for screening or taking care of suspected or confirmed COVID-19 patients, and (iii) citizens without identifiable risk of contracting SARS-CoV-2. We hypothesized that these three groups of population specifically have different roles in the control of community transmission of SARS-CoV-2 in Taiwan (see **Supplementary Table**). In addition, because previous studies demonstrated a cross-reactive antibody response between SARS-CoV-2 and severe acute respiratory syndrome coronavirus (SARS-CoV) infection (20, 21), individuals with virologically confirmed SARS-CoV infection in 2003 were separately invited and analyzed to avoid overestimating the seroprevalence among study populations from cross-reactivity between the two genetically close virus (22). Our goal was first to estimate the true seroprevalence among populations with different SARS-CoV-2 exposure risks. The second goal was to understand the anti-SARS-CoV-2 antibody response among individuals with SARS in 2003.

### Policies Against the COVID-19 Pandemic in Taiwan

At the beginning of the COVID-19 outbreak from December 2019, Taiwan had implemented proactive measures against this novel virus. The containment measures and policies were adjusted dynamically according to the severity and global situations of the epidemic. Strategies in Taiwan were integrated and composed of three main components: border control, healthcare system response, and public engagement (see **Supplementary Table**). Before April 30, travel restrictions were applied to countries with known or suspected widespread community transmission of SARS-CoV-2. The number of people entering Taiwan decreased significantly from 2,262,692 in January to 22,822 in April, with a total of 3,638,171 people between January 1st and April 30. All entry travelers received a COVID-19 Health Declaration and Home Quarantine Notice to self-report if they experienced any COVID-19-compatible symptoms before going through the immigration. Febrile or symptomatic travelers identified at the airport or during the 14-day quarantine period will undergo a SARS-CoV-2 qRT-PCR test. Confirmed COVID-19 patients with qRT-PCR test will be admitted to a hospital isolation room to prevent the spread of the virus in the community. HCWs in hospitals or nursing facilities comply with strict infection prevention and control guidelines recommended by Taiwan Centers for Disease Control. Personal protective equipment (PPE) are fully supplied for first-line HCWs, especially for the quarantine ward or intensive care unit (ICU) staffs taking care of suspected or confirmed COVID-19 patients. All citizens are encouraged to wear a mask, maintain hand hygiene and social distancing, and avoid mass gathering (23). In addition to purchasing vaccines with emergency use authorization from abroad, Taiwan government also supports SARS-CoV-2 vaccine development by local manufacturers. By April 30, a total of 62,844 individuals suspected of SARS-CoV-2 infection were reported to the Taiwan Centers for Disease Control and received at least one qRT-PCR test for the diagnosis of COVID-19. Among them, 429 COVID-19 patients were diagnosed, including 343 imported cases.

## Materials and Methods

### Hospital Settings, Study Design, and Participant Enrollment

Two hospitals in northern Taiwan participated in this cross-sectional seroprevalence study. The National Taiwan University Hospital (NTUH), located in Taipei, is a 2,700-bed teaching hospital that provides both primary and tertiary care. The NTUH Hsin-Chu Biomedical Park Branch (NTUH HBP Branch) is a 475-bed community hospital in Hsinchu County and is about 62 km from NTUH. The study was conducted on June 29 to July 12, 2020, in NTUH and on July 25, 2020, in NTUH HBP branch. Study approvals from the institutional review board were obtained from NTUH (202004128RINB) and NTUH HBP Branch (202007007RIPB).

The seroprevalence of anti-SARS-CoV-2 antibodies was determined in three target populations: (i) Population P: patients with epidemiological risk of SARS-CoV-2 exposure (returned travelers or foreigners for international business; certain occupations such as HCWs not responsible for screening or taking care of COVID-19 patients, airport staffs, flight crew or local tour guide; having contact with known or suspected COVID-19 patient; and cluster phenomenon of infection such as presence of fever or airway symptom in a family or workplace) and compatible symptom who visited epidemic outpatient clinic or emergency department for COVID-19 screening during January 21 to April 30, 2020 (in NTUH). Detailed information about specific epidemiological risk exposure is shown in **Table 1**; (ii) Population H: HCWs responsible for screening or taking care of suspected or confirmed COVID-19 patients since January 21 to April 30, 2020, which included staffs in the emergency department, epidemic outpatient clinic, COVID-19 quarantine ward or ICU, and clinical laboratory (during that period, 16 qRT-PCR-confirmed COVID-19 patients, including six referred from other hospitals after diagnosis, were hospitalized in NTUH with a total of 480 patient-days in the general isolation ward and 74 patient-days in the ICU (in NTUH]); (iii) Population C: adult citizens who were eligible for a hepatitis screening program on July 25, 2020 (in NTUH HBP Branch). Among patients with the risk of SARS-CoV-2 exposure and who received COVID-19 screening at NTUH during the study period, 10 qRT-PCR-confirmed COVID-19 patients were not included because we aimed to identify undiagnosed COVID-19 patients under the quarantine and surveillance policy in Taiwan. Besides the three aforementioned populations for the seroprevalence study, we also enrolled individuals with virologically documented SARS-CoV infection in 2003 to clarify the cross-reactivity between anti-SARS-CoV and anti-SARS-CoV-2 antibody response. Eligible individuals were invited by postal mail, telephone call, and poster. Informed consent was obtained from all adult participants or from their legal representatives for participants younger than 20 years. Sex and age of all participants were recorded at the time of blood sampling. Clinical information including initial presentation, date of symptom onset, and epidemiological risk for SARS-CoV-2 exposure of screened patients was retrieved from electronic medical records of the study hospital.

**Table 1:** Clinical characteristics and epidemiological risk factors of contracting severe acute respiratory syndrome coronavirus 2 (SARS-CoV-2), of the 499 participants who visited the study hospital for coronavirus disease 2019 (COVID-19) screening.

Parameter	Patient with exposure risk (n = 499)	
Age	40.0 ± 15.1	
Male gender	178	(35.7%)
Duration from symptom onset to serological testing, days	122.8 ± 28.5	
Clinical symptoms		
Fever	206	(41.3%)
Cough	264	(52.9%)
Sore throat	188	(37.7%)
Dyspnea	50	(10.0%)
Fatigue	19	(3.8%)
Diarrhea	55	(11.0%)
Loss of taste or smell	8	(1.6%)
Epidemiological risk factors		
Travel history	335	(67.1%)
Asia		
China	77	(15.4%)
Japan	59	(11.8%)
South Korea	11	(2.2%)
Singapore	12	(2.4%)
Hong Kong	24	(4.8%)
Macau	9	(1.8%)
Thailand	8	(1.6%)
Vietnam	9	(1.8%)
Other Asian countries^a^	27	(5.4%)
America		
USA	28	(5.6%)
Canada	6	(1.2%)
Other American countries^b^	3	(0.6%)
Europe		
England	17	(3.4%)
Germany	5	(1.0%)
France	6	(1.0%)
Spain	3	(0.6%)
Other European countries^c^	21	(4.2%)
Africa	5	(1.0%)
Australia	5	(1.0%)
Occupation		
Healthcare workers	77	(15.4%)
School teacher or student	14	(2.8%)
Taxi driver	3	(0.6%)
Service industry	24	(4.8%)
Aviation industry	6	(1.2%)
Contact		
Confirmed COVID-19 patient	13	(2.6%)
Suspected COVID-19^d^	19	(3.8%)
Cluster		
Household	26	(5.2%)
Workplace/school	24	(4.8%)
Public place/transportation	14	(2.8%)
Received qRT-PCR test for SARS-CoV-2	447	(89.6%)

a Include Philippines, Malaysia, Indonesia, Dubai, Myanmar, Cambodia, India, Turkey, and Nepal.

b Include Chile, Cuba, and Panama.

c Include Ireland, Portugal, Italy, Netherlands, Belgium, Iceland, Hungary, Austria, Czech Republic, Switzerland, Norway, Finland, and Ukraine.

d Include those who had travel history from a COVID-19 epidemic area or those who was considered a probable case for COVID-19 infection.

### Sample Collection and Anti-SARS-CoV-2 Antibody Testing

After obtaining the informed consent, 3 ml of peripheral venous blood from each participant was collected. The serum of the collected blood samples was stored at −20°C before testing. We used two automated chemiluminescent immunoassays to detect anti−SARS−CoV−2 antibodies. The Roche Elecsys^®^ Anti−SARS−CoV−2 assay is an electrochemiluminescence immunoassay using the recombinant nucleocapsid protein (N protein) for the detection of total antibodies (including IgG) against SARS−CoV−2 with Cobas e immunoassay analyzers (Roche Diagnostics Basel, Switzerland) (Roche test) (18, 24). The Abbott SARS-CoV-2 IgG assay is a chemiluminescent microparticle immunoassay that qualitatively detects IgG antibodies on the SARS-CoV-2 N protein in human serum and plasma using the ARCHITECT i System (Abbott Laboratories, IL, USA) (Abbott test) (19, 25). Sample with a reported cutoff index (COI) greater than 1.00 in Roche test and index sample/calibrator (index S/C) greater than 1.40 in Abbott test was considered as a positive result of the test, respectively.

The serum of all participants with known SARS infection history and the serum of participants in the seroprevalence study with a positive result for anti-SARS-CoV-2 antibodies by either the Roche test or Abbott test were further tested with three qualitative lateral flow immunoassays (LFIA) for anti-SARS-CoV-2 antibodies: (i) Wondfo SARS-CoV-2 Antibody Test (Guangzhou Wondfo Biotech Co., Ltd., China) (Wondfo test), (ii) ASK COVID-19 IgG/IgM Rapid Test (TONYAR Biotech Inc., Taiwan) (ASK test), and (iii) Dynamiker 2019-nCoV IgG/IgM Rapid Test (Dynamiker Biotechnology [Tianjin] Co., Ltd., China) (Dynamiker test) (17). The ASK test and Dynamiker test detect either anti-SARS-CoV-2 IgG and IgM antibodies separately, and the Wondfo test detects total antibodies against SARS-CoV-2 within 5–15 min. The viral protein labeled was the SARS-CoV-2 spike protein (S protein) in the Wondfo and ASK tests and SARS-CoV-2 N protein in the Dynamiker test. A weakly positive result (any shade of color in the test lines) of an antibody rapid testing was considered positive according to the manufacturers’ instructions. In our previous studies, the three serological tests of lateral flow immunoassay method showed comparable performance in detecting anti-SARS-CoV-2 antibodies but no cross-reactivity with autoantibodies or antibodies against other coronavirus or non-coronavirus than serological tests of chemiluminescent immunoassay method (17, 26). In addition, a serological test for cytomegalovirus (CMV) IgM/IgG antibodies was performed to detect possible cross-reactivity between anti-SARS-CoV-2 and anti-CMV antibodies to increase diagnostic specificity. Finally, we used *recom*Line SARS-CoV-2 IgG (MIKROGEN Diagnostik GmbH, Neuried, Germany), a line immunoassay specifically identifying antibodies against the individual antigens of the coronaviruses (nucleocapsid protein [NP], receptor binding domain [RBD], and S1 from SARS-CoV-2 as well as NP for human coronavirus 229E, NL63, OC43, and HKU1), as a confirmatory test to clarify the initial results of the Roche and Abbott tests among participants in the seroprevalence study (27).

### Statistical Analysis and Case Number Estimate

**Figure 1:**
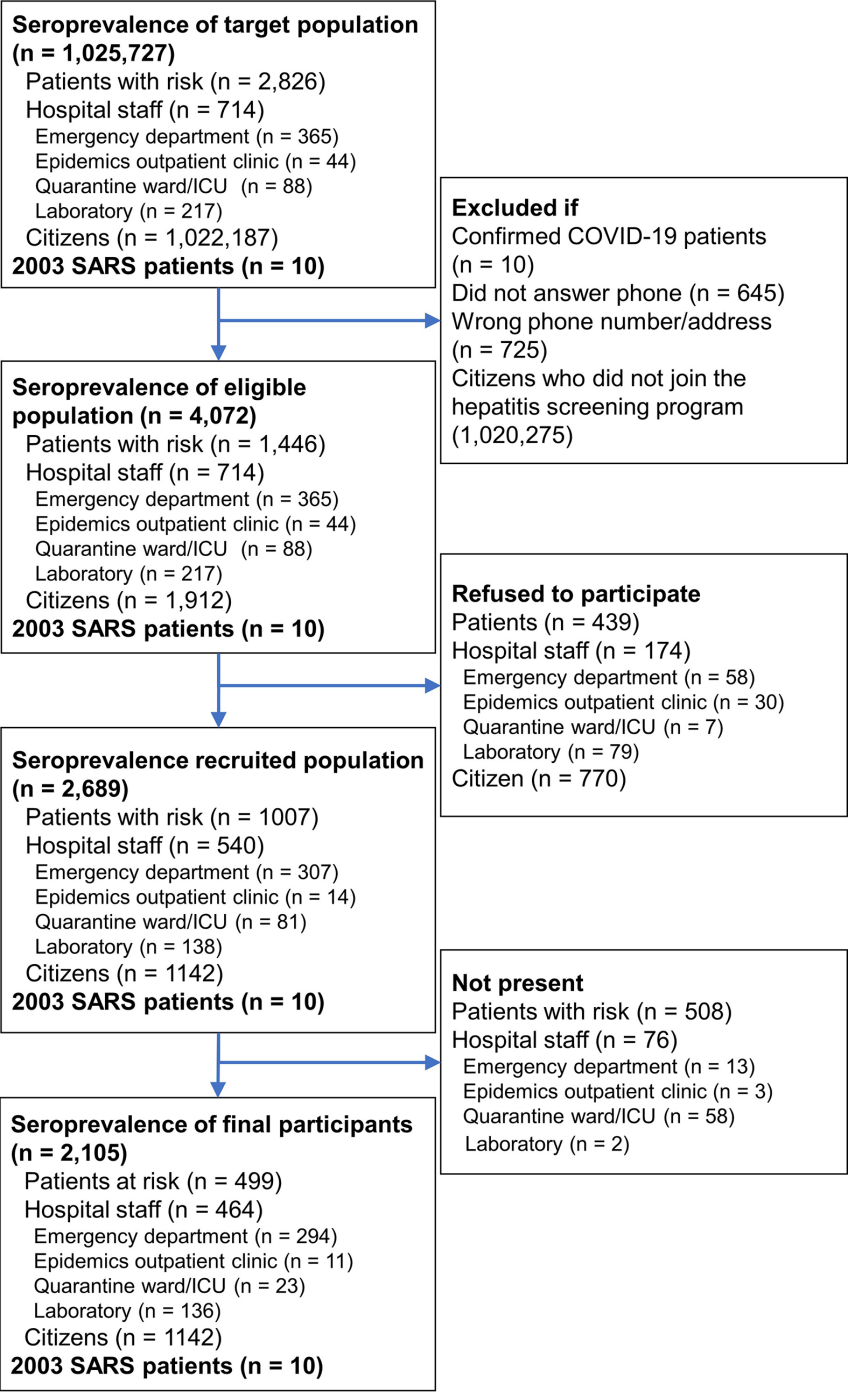
Participants’ enrollment flowchart.

We calculated means and standard deviations (SDs) for age variables and percentages for categorical variables. Binomial proportion confidence interval (CI) was used to estimate the range of seroprevalence. The required sample size was estimated using a single proportion population formula (28) with a 95% confidence level and 1% margin of error. The population size for Population P and Population H were retrieved from electronic medical records and personnel registry of the study hospital (NTUH) and were 2,826 and 714, respectively (**Figure 1**). The population size of 1,022,187 for Population C was the total population of HsinChu City and HsinChu County at the end of 2020 according to government statistics (29, 30). Expected seroprevalence for Population P, Population H, and Population C were estimated to be 3, 2 and 1% according to the seroprevalence reports at the time of study (31–33). The minimum number of participants for Population P, Population H, and Population C were estimated at 802, 367, and 381, respectively. Data were analyzed using SPSS for Windows (IBM SPSS Statistics v26).

## Results

In the seroprevalence study, 5,452 individuals were initially invited. Individuals who had previously confirmed SARS-CoV-2 infection, failed to be contacted, refused to participate, or did not show up during the study period were excluded. Finally, a total of 2,105 individuals, including 499 patients with risk for COVID-19 infection (Group P), 464 hospital staffs (Group H), and 1,142 citizens (Group C), provided consent and were tested for anti-SARS-CoV-2 antibodies (**Figure 1**). Of the 499 Group P participants, 308 (61.7%) had travel history from China (15.4%), other Asian countries (26.5%), European countries (10.4%), American countries (7.4%), African countries (1.0%), and Australia (1.0%) within 2 weeks of symptom onset. qRT-PCR was performed in 447 (89.6%) of the Group P participants and all were negative for SARS-CoV-2. Age and male sex percentage were 40.0 ± 15.1 years and 35.7%, respectively. Duration from symptom onset to serological testing in Group P participants was 122.8 ± 28.5 days (**Table 1**). Age and male sex percentage were 37.4 ± 10.8 years and 35.0% for Group H participants, 49.5 ± 12.6 years and 50.2% for Group C participants, and 50.6 ± 10.7 years and 40.0% for Group S participants, respectively.

**Figure 2:**
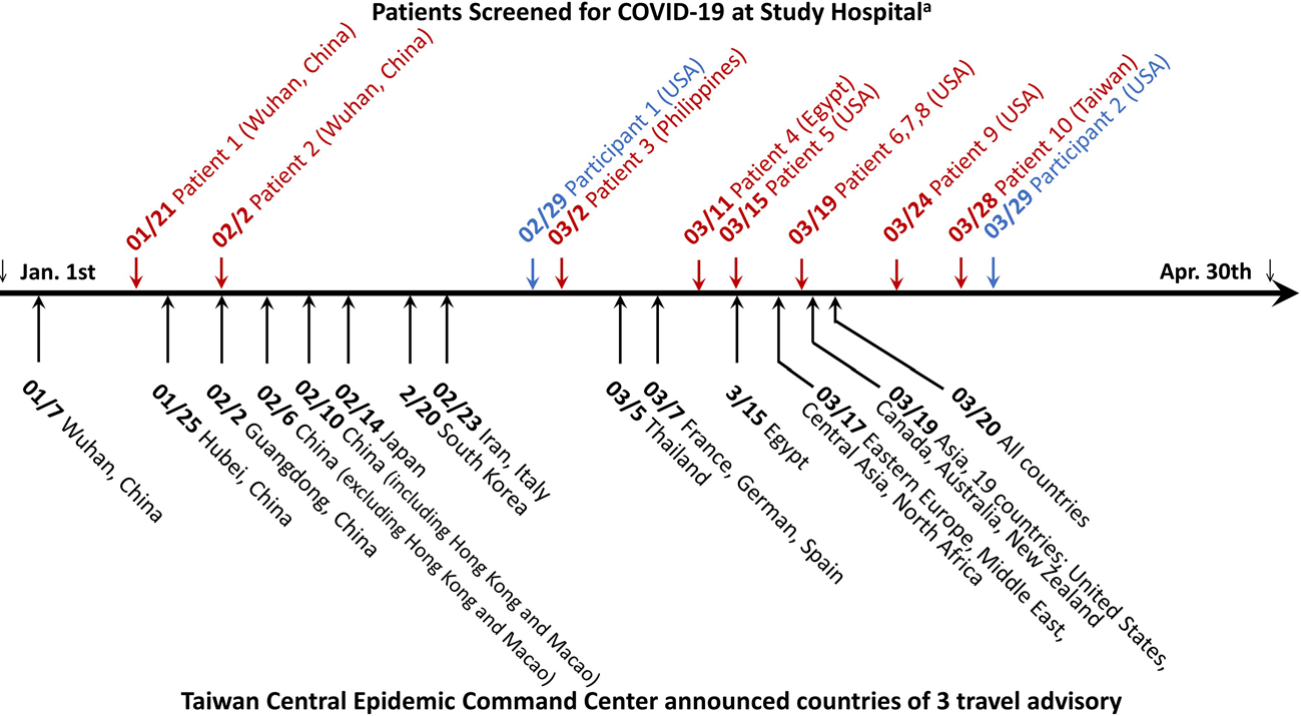
Timeline of COVID-19 patient diagnosed at National Taiwan University Hospital and travel advisory announced by Taiwan Central Epidemic Command Center between January and April 2020. The diagnosis of COVID-19 was achieved either by a positive qRT-PCR testing result at initial screening visit in the emergency department or epidemic outpatient clinic (letters in red color) or retrospectively by serological test in this study (letters in blue color). The number-slash-number before COVID-19 patient denotes the latest date of leaving an area or country reporting COVID-19 outbreak for the 11 imported cases and the date of COVID-19 diagnosis for the one indigenous case. The city or country name indicates the travel location of the 11 imported cases, except one indigenous case who did not have travel history abroad.

In the seroprevalence study including 2105 Groups P, H and C participants, a total of eight participants were positive for anti-SARS-CoV-2 antibodies in either the Roche test (six participants) or Abbott test (four participants) (**Table 2**). The sera of eight participants were further tested with three LFIAs, line immunoassay, and anti-CMV IgG/IgM antibodies. Among them, only two participants were further confirmed as true positive with the line immunoassay. Both of them were returning travelers from the United States on February 29 and March 29, respectively (**Figure 2**). They had fever and airway symptom but negative results on multiple screening qRT-PCR assay during the quarantine period. Another six participants were judged stringently as false positive due to a negative result for anti-SARS-CoV-2 S1, RBD, or N-protein antibodies in line immunoassay. LFIAs for the six participants’ sera were all negative for anti-SARS-CoV-2 antibody response and were compatible with the results in the line immunoassay. Of the six sera interpreted as false positive result for anti-SARS-CoV-2 antibodies, cross-reactivity with seasonal human coronavirus and possibly with anti-CMV IgM/IgG antibody was observed in five participants.

**Table 2:** Summary of eight participants with positive results from either Roche Elecsys^®^ Anti−SARS−CoV−2 test (Roche) or Abbott SARS-CoV-2 IgG assays (Abbott) in a seroprevalence survey (n = 2105) conducted from June 25 to July 29, 2020.

No.	Cohort of participants	1st/2nd tests^a^		Lateral flow immunoassay			*recom*Line SARS-CoV-2 IgG Line immunoassay							CMV IgM/IgG antibody	Interpretation for SARS-CoV-2 antibody testing
		Roche (COI)	Abbott (index S/C)	ASK(IgM/IgG)	Wondfo (T)	Dynamiker (IgM/IgG)	S1 SARS-2	RBD SARS-2	NP SARS-2	NP HKU1	NP OC43	NP NL63	NP 229E		
1	Group P	**134.6/ 133.1**	**6.17/ 6.17**	**+/+**	**+**	**+/+**	**+**	**+**	**+**	**+**	**+**	**+**	**+**	−/−	**Positive**
2		**112.7/ 111.5**	**5.92/ 5.97**	**+/+**	**+**	**+/+**	**+**	**+**	**+**	**+**	**+**	**+**	**+**	−/−	**Positive**
3		**2.02/ 2.05**	0.03/ 0.03	−/−	−	−/−	−	−	−	**+**	**+**	**+**	**+**	−**/+**	False positive
4	Group H^b^	**1.46/ 1.46**	0.02/ 0.02	−/−	−	−/−	−	−	−	−	−	−	−	−/−	False positive
5		**1.26 1.29**	0.07/ 0.08	−/−	−	−/−	−	−	−	**+**	**+**	−	−	−**/+**	False positive
6		**2.51/ 2.47**	0.04/ 0.05	−/−	−	−/−	−	−	−	**+**	**+**	**+**	−	−/−	False positive
7		0.153/0.163	**1.9/ 1.87**	−/−	−	−/−	−	−	−	**+**	**+**	**+**	**+**	−**/+**	False positive
8	Group C	0.076/0.076	**1.42/ 1.50**	−/−	−	−/−	−	−	−	**+**	−	−	−	−**/+**	False positive

SARS-CoV-2, severe acute respiratory syndrome coronavirus 2; index S/C, index sample/calibrator; COI, cutoff index; T, total antibodies; S1, spike subunit 1; RBD, receptor-binding domain; NP, nucleocapsid protein; SARS-2, SARS-CoV-2; HKU1, human coronavirus-HKU1; OC43, human coronavirus-OC43; NL63, human coronavirus-NL63; 229E, human coronavirus-229E; CMV, cytomegalovirus; Group P, patients with epidemiological risk of SARS-CoV-2 exposure; Group H, healthcare workers; Group C, healthy adult citizens in Hsinchu area.

a All the tests were performed twice. Sample with COI ≥1.00 in Roche test and index S/C ≥1.40 in Abbott test was considered a positive result and was highlighted in bold.

b All are emergency department staffs.

**Figure 3:**
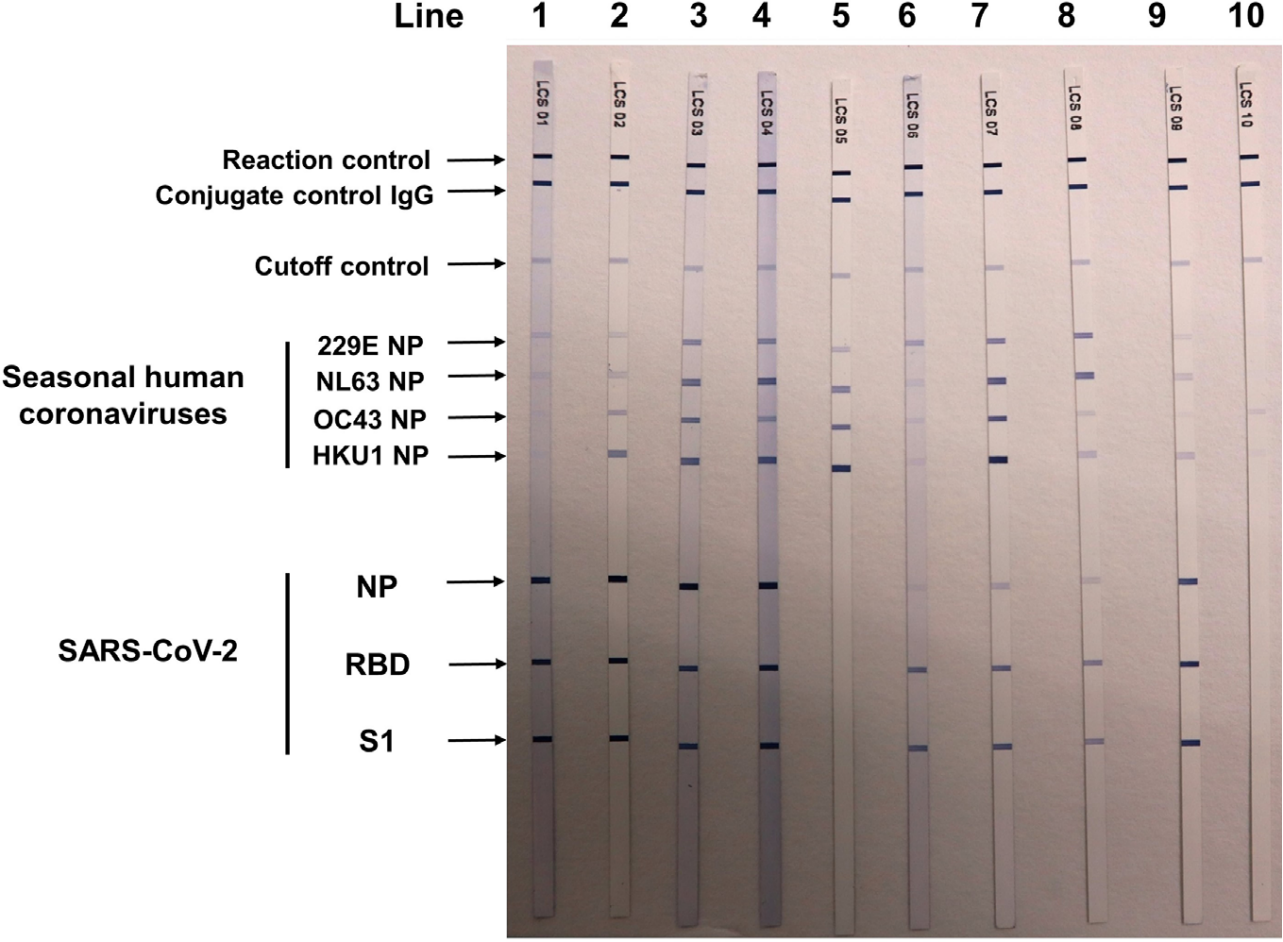
Results of *recom*Line SARS-CoV-2 IgG [Avidität] line immunoassay (MIKROGEN Diagnostik GmbH, Neuried, Germany) for two qRT-PCR-confirmed COVID-19 patients and eight participants in the seroprevalence survey. Lines 1 and 2: two patients with qRT-PCR-confirmed COVID-19 patients treated at National Taiwan University Hospital (NTUH). Lines 3–5: participant nos. 1 and 2 and 6 in **Table 2**. Lines 6–9: four healthcare workers (nos. 1, 5, 6, and 8 in **Table 3**) with SARS-CoV infection in 2003. Line 10: a patient visited NTUH with acute respiratory infection, and triplicate qRT-PCR testings for SARS-CoV-2 from respiratory samples were negative. NP, nucleocapsid protein; RBD, receptor-binding domain; S1, spike subunit 1.

Ten individuals with previous SARS infection history participated in this study to help evaluate the cross-reactive antibody response between SARS-CoV-2 and SARS-CoV infections (Group S). All of the 10 Group S participants were incumbent or retired HCWs. None of them took care of or screened patients with confirmed or suspected COVID-19 infection before testing. They also had no known epidemiological risk of contracting SARS-CoV-2 in the past half year before study enrollment. Among the 10 Group S participants, eight (80.0%) were positive for anti-SARS-CoV-2 antibodies in the Roche test, but none were positive in the Abbott test and the three LFIA tests. Five Group S participants were positive for anti-SASR-CoV-2 S1 and RBD antibodies in the line immunoassay. One Group S participant who showed high cutoff index (COI) value (71.97 and 73.22, respectively; COI ≥1.0 considered as reactive) was positive for anti-SASR-CoV-2 S1, RBD, and NP antibodies in the line immunoassay (**Table 3**). **Figure 3** demonstrates the results of the *recom*Line SARS-CoV-2 IgG line immunoassay for two qRT-PCR-confirmed, hospitalized COVID-19 patients and eight participants in this study.

**Table 3:** Summary of results of different antibody assays from 10 healthcare workers with virologically-documented SARS-CoV infection in 2003.

No.	1st/2nd tests^a^		Lateral flow immunoassay			*recom*Line SARS-CoV-2 IgG Line immunoassay							CMV IgM/IgG antibody
	Roche (COI)	Abbott (index S/C)	ASK (IgM/IgG)	Wondfo (T)	Dynamiker(IgM/IgG)	S1 SARS-2	RBD SARS-2	NP SARS-2	NP HKU1	NP OC43	NP NL63	NP 229E	
1	**8.3/ 8.14**	0.06/ 0.07	−/−	−	−/−	**+**	**+**	−	−	−	−	**+**	−**/+**
2	0.404/0.41	0.87/ 0.86	−/−	−	−/−	**+**	**+**	−	−	−	**+**	−	−/−
3	**1.56/ 1.54**	0.05/ 0.05	−/−	−	−/−	−	−	−	−	−	−	−	−/−
4	**1.46/ 1.46**	0.09/ 0.09	−/−	−	−/−	**+**	**+**	−	−	−	−	**+**	−**/+**
5	**11.84/ 11.5**	0.38/ 0.36	−/−	−	−/−	**+**	**+**	−	**+**	**+**	**+**	**+**	−**/+**
6	**10.36/ 9.98**	0.06/ 0.07	−/−	−	−/−	**+**	**+**	−	−	−	**+**	**+**	−/**+**
7	0.024/ 0.236	0.05/ 0.05	−/−	−	−/−	−	−	−	−	−	−	−	−**/+**
8	**71.97/ 73.22**	0.77/ 0.74	−/−	−	−/−	**+**	**+**	**+**	−	−	−	−	−**/+**
9	**6.44/ 6.54**	0.18/ 0.18	−/−	−	−/−	−	−	−	−	−	−	−	−**/+**
10	**2.67/ 2.81**	0.76/ 1.20	−/−	−	−/−	−	−	−	−	−	−	−	−**/+**

Index S/C, index sample/calibrator; COI, cut-off index; T, total antibodies; S1, spike subunit 1; RBD, receptor-binding domain; NP, nucleocapsid protein; SARS-2, SARS-CoV-2; HKU1, human Coronavirus-HKU1; OC43, human Coronavirus-OC43; NL63, human Coronavirus-NL63; 229E, human Coronavirus-229E; CMV, cytomegalovirus.

a All the tests were performed twice. Sample with COI ≥1.00 in Roche test and index S/C ≥1.40 in Abbott test was considered a positive result and was highlighted in bold.

**Figure 4:**
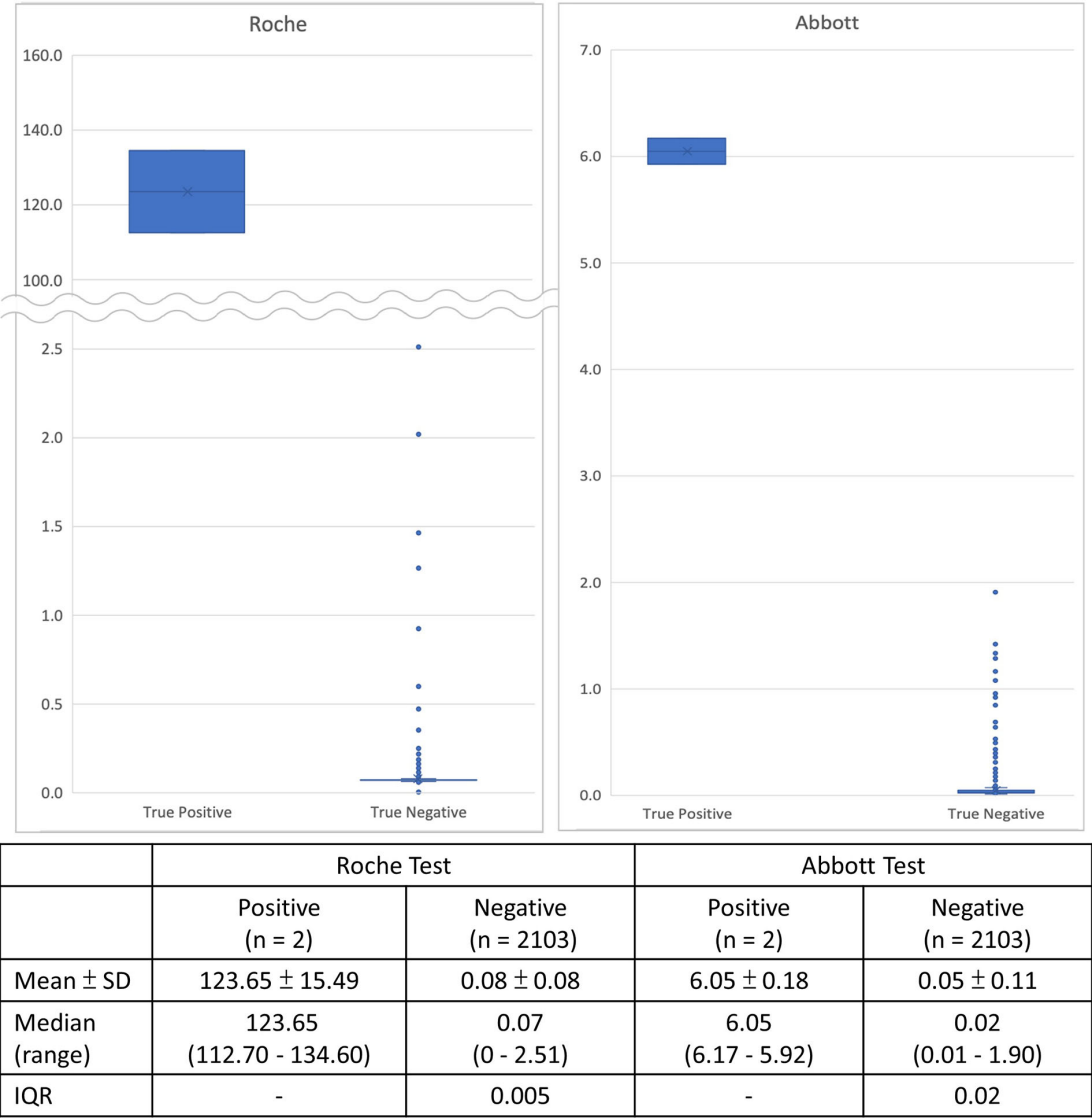
Distribution of serological test results of the 2,105 seroprevalence study participants of the two automated chemiluminescent immunoassays, stratified by final positive and negative interpretation from combining the results of the *recom*Line SARS-CoV-2 IgG line immunoassay and the three lateral flow immunoassays.

Results of serological testing for anti-SARS-CoV-2 antibodies are summarize in **Table 4**. The crude seroprevalence among the three study groups were 0.6% (95% CI, 0.12–1.75%) in Group P participants, 0.86% (95% CI, 0.24–2.19%) in Group H participants, and 0.09% (95% CI, 0–0.49%) in Group C participants. If the six participants interpreted as having false positive testing result were exclude, the stringently judged seroprevalence among the three study groups, therefore, was 0.4% (95% CI, 0.05–1.44%) in Group P participants, 0% (95% CI, 0–0.79%) in Group H participants, and 0% (95% CI, 0–0.32%) in Group C participants. Finally, data about the distribution of the Roche and Abbott test results of the 2,105 seroprevalence study participants, stratified by stringently judged two positive and 2,103 negative results, is shown in **Figure 4**.

**Table 4:** Summary of sources of participants and corresponding seroprevalence for coronavirus disease 2019 using Roche Elecsys^®^ Anti−SARS−CoV−2 test and Abbott SARS-CoV-2 IgG assay.

Population	No. of participants	No. of participants with positive results for SARS-CoV-2 antibodies^a^	Crude seroprevalence, % (95% CI)	No. of participants with judged true-positive results for SARS-CoV-2 antibodies^b^	Stringently judged seroprevalence, % (95% CI)
Seroprevalence study population^c^	2,105	8	0.38 (0.16–0.75)	2	0.10 (0.01–0.34)
Patients with exposure risk^d^	499	3	0.60 (0.12–1.75)	2	0.40 (0.05–1.44)
Healthcare workers	464	4	0.86 (0.24–2.19)	0	0 (0.00–0.79)
Emergency department	294	4	1.36 (0.37–3.45)	0	0 (0.00–1.25)
Epidemics outpatient clinic	11	0	0 (0.00–28.49)	0	0 (0.00–28.49)
Quarantine medical ward	12	0	0 (0.00–26.46)	0	0 (0.00–26.46)
Intensive care unit^e^	11	0	0 (0.00–28.49)	0	0 (0.00–28.49)
Laboratory	136	0	0 (0.00–2.68)	0	0 (0.00–2.68)
Citizens (Hsinchu area)^f^	1,142	1	0.09 (0.00–0.49)	0	0 (0.00–0.32)
SARS-CoV infection in 2003^g^	10	8	80.0 (44.39–97.48)	0	0 (0.00–30.85)

a Indicates positive results by either Roche Elecsys® Anti−SARS−CoV−2 test or Abbott SARS-CoV-2 IgG assays.

b Judged by combining the results of additional tests including three anti-SARS-CoV-2 lateral flow immunoassays, recomLine SARS-CoV-2 IgG line immunoassay, and anti-cytomegalovirus antibody test.

c Patients who had no virologically documented SARS-CoV-1 infection in 2003 and coronavirus disease 2019 infection in 2020.

d Patients with risk of SARS-CoV-2 exposure and clinical symptom who visited the emergency department or quarantine outpatient clinics at National Taiwan University Hospital for COVID-19 diagnosis but were tested negative in laboratory testing or excluded directly by clinical evaluation between January 21 and April 30, 2020.

e Only one designated intensive care unit that admitted severe cases of coronavirus disease 2019 was enrolled in this study.

f A total of 1,912 citizens in Hsinchu area were enrolled in a hepatitis screening program conducted in July, 2020. Among them, 1,142 participated in the seroprevalence survey for anti-SARS-CoV-2 antibody and signed the informed consent form of the study.

g All were healthcare workers in 2003.

## Discussion

Our study simultaneously evaluated the seroprevalence of the antibody response to SARS-CoV-2 in three different populations in northern Taiwan, with the sample collection period from late June through mid-July 2020. After strictly excluding false-positive results including those from participants with prior SARS infection in 2003, we found that the seroprevalence was 0.4% among participants who had epidemiological risk of SARS-CoV-2 exposure but were negative for COVID-19 diagnosis in hospital screening during the early stage of the COVID-19 outbreak in Taiwan. None of the HCWs and citizen participants had positive anti-SARS-CoV-2 antibody testing result in this study. The risk-stratified seroprevalence estimates are the first report from Taiwan that have successfully staved off the COVID-19 pandemic with timely implementation of effective preventive measures against the invasion of SARS-CoV-2.

Immediately after the outbreak of COVID-19 in China, Taiwan authority implemented strict public health interventions and stepwise announced international travel restrictions to prevent the spread of SARS-CoV-2 into the community. Many hospitals set up outdoor or specialized screening clinics in outpatient clinics or emergency departments to prevent nosocomial outbreak of COVID-19 in the hospital (34). From January 21 through April 30, a total of 2,826 patients with epidemiological risk for COVID-19 infection received clinical or laboratory screening in NTUH; among them 10 were diagnosed with COVID-19 infection by positive qRT-PCR test. Two COVID-19 cases out of the 499 Group P patients were additionally diagnosed by serological testing in this study. If the remaining 2,317 patients who did not participate in this study had the same 0.4% seroprevalence as of the 499 Group P participants, additional nine COVID-19 patients could be retrospectively identified by serological testing, suggesting that the exact COVID-19 patient number among the 2,826 screened patients between January and April 2020 would be 21 rather than 10, corresponding to 0.74% (21/2,826) COVID-19 disease prevalence among symptomatic patients with epidemiological risk. The detection sensitivity of the current screening strategy combining clinical evaluation and laboratory qRT-PCR testing therefore might only be 47.6% (10/21). The projected COVID-19 prevalence was much higher than the prevalence based on clinical and viral nucleic acid assay screening. These undiagnosed COVID-19 infections were likely due to the low viral load below the detection limit of the qRT-PCR testing, or with atypical or mild symptoms in which COVID-19 infection was not suspected by the visiting physician at the time of screening.

The outbreak of SARS-CoV-2 infection and consequently the emergence of a tremendous number of COVID-19 patients led to medical service system failure and staff burnout (35, 36). A high seroprevalence of up to 5–23% has been reported in many countries, especially in countries or areas experiencing severe community outbreaks of COVID-19 (14, 37–40). The HCWs responsible for screening or taking care of COVID-19 patients were even at a higher risk of contracting SARS-CoV-2, especially in areas with a short supply of PPE (41, 42). In contrast, without community transmission and only a limited number of COVID-19 cases, Taiwan attracts worldwide attention with respect to the amazing performance of containing the COVID-19 pandemic. Although an undetected circulation of SARS-CoV-2 in the community has been suspected, our study confirmed the extremely low seroprevalence of SARS-CoV-2 in Taiwan under the current epidemic controlling strategies. However, clinical and viral nucleic acid-based laboratory screening systems might diagnose less than one-half of symptomatic COVID-19 patients, as demonstrated in a previous study (43), and in the current study. It is plausible that 429 confirmed COVID-19 patients in Taiwan by April 30 indicate that additional 465 undiagnosed symptomatic COVID-19 patients escaped from the current airport and hospital screening system deduced from the data analyses in this study. Those undiagnosed symptomatic COVID-19 patients as well as unrecognized asymptomatic COVID-19 patients inevitably spread the SARS-CoV-2 into the healthcare system and community. The low seroprevalence in hospital staffs supports the effectiveness of implementing strict infection control measures and providing adequate PPE for first-line HCWs in the prevention of nosocomial transmission of the SARS-CoV-2. The low seroprevalence in our citizens demonstrates the importance of establishing physical (mask-wearing, social distancing, avoiding mass gathering) and chemical barriers (handwashing, alcohol scrub) in the interruption of SARS-CoV-2 transmission in communities.

In this study, 61.7% of patients who visited NTUH for SARS-CoV-2 infection screening had travel history 2 weeks prior to symptom onset from countries reporting confirmed COVID-19 cases. The low SARS-CoV-2 seroprevalence among these patients implies the low influx pressure of the virus from abroad. It reasonably reflects the extent and severity of the COVID-19 pandemic in the early emerging period of this novel virus. It is considered a warning, however, to the current success of COVID-19 control in Taiwan. As the severity of the COVID-19 pandemic rises globally, the risk of SARS-CoV-2 invasion and spread into Taiwan with returning travelers or international business travelers is inevitably increasing, especially from those with asymptomatic infection with prolonged communicability period and virus shedding (44–47). The risk will be even higher after Taiwan opens the border to revitalize international business to boost the economy. Additional strategies, such as a screening policy after 14 days of quarantine, should be considered to prevent the SARS-CoV-2 invasion in Taiwan.

The potential cross-reactive antibody response between SARS-CoV-2 and SARS-CoV among recovered SARS patients long after an infection was an important finding in this study. Previous studies showed that the anti-SARS-CoV IgG and neutralizing antibodies decreased markedly 2 years after the infection and were detected in only 8.7% of patients 6 years after recovery from SARS infection (48, 49). SARS-CoV-specific peripheral memory B cell response was undetectable, but memory T cell responses could be identified in 60.9% of recovered SARS patients 6 years after infection (49). One possible explanation is the increased detection sensitivity of current serological tests compared to that of ELISA kits used in previous studies (49–51). Nonetheless, our study suggested the possibility of the prolonged existence of anti-SARS-CoV antibodies after 17 years of infection causing a false-positive testing result from the cross-reactive antibody response between the two viruses with close genetic composition (52, 53). Therefore, in the seroprevalence study for COVID-19 infection in the areas with prior SARS outbreak, false-positive testing results should be considered among participants with virologically confirmed SARS infection.

This study has some limitations. First, this was a two-center study conducted in northern Taiwan. Thus, a nationwide seroprevalence study to confirm our study findings is imperative. Second, the inadequate case number of a seroprevalence study in areas with low disease prevalence might have failed to detect sporadic COVID-19 infection in the community and healthcare system. It was especially important in the estimation of the seroprevalence among patients with epidemiological risk of SARS-CoV-2 exposure in this study. Third, although both the Roche and Abbott tests are based on antibodies against SARS-CoV-2 N protein, the relative higher false-positive rate in Roche test was not further investigated in this study. Fourth, we did not test the anti-SARS-CoV antibodies in patients with virologically-documented SARS-CoV infection in 2003. It is not known whether the cross-reactivity was caused by anti-SARS-CoV antibody after 17 years since SARS-CoV infection. Fifth, returning or foreign travelers without symptoms were not included in this study. It was impossible to detect asymptomatic infection, and therefore, the threat from influx pressure of SARS-CoV-2 was underestimated. Finally, asymptomatic infection with transient or absent antibody response to SARS-CoV-2 infection remains theoretically plausible. Therefore, extent of SARS-CoV-2 transmission in the community might be further underestimated (54).

## Conclusion

In conclusion, rigorous exclusion of false-positive testing results is imperative for an accurate estimate of seroprevalence and disease burden in countries with low COVID-19 disease prevalence and the existence of outbreaks of SARS in 2003. The overall SARS-CoV-2 seroprevalence was extremely low in Taiwan, reflecting the early success of nationwide containment strategies. However, with the increasing severity of the global COVID-19 pandemic and virus burden, additional countermeasures based on current strategies such as the establishment of adequate herd immunity by vaccination to prevent the invasion and spread of SARS-CoV-2 in Taiwan should be considered.

## Data Availability Statement

The datasets used and/or analysed during the current study are available from the corresponding author on reasonable request.

## Ethics Statement

The study was conducted on June 29 to July 12, 2020, in NTUH and on July 25, 2020, in NTUH HBP branch. Study approvals from the institutional review board were obtained from NTUH (202004128RINB) and NTUH HBP Branch (202007007RIPB). The patients/participants provided their written informed consent to participate in this study.

## Author Contributions

W-PT, J-LW, C-CW, and P-RH designed the research. K-TK, C-HL, M-YC, Y-FL, C-JY, C-HH, and S-YC performed the research and analyzed the data. W-PT, J-LW, and S-YC wrote the manuscript. All authors contributed to the article and approved the submitted version.

## Funding

The study was funded by the National Taiwan University Hospital (grant numbers: NTUH 109-P12).

## Conflict of Interest

The authors declare that the research was conducted in the absence of any commercial or financial relationships that could be construed as a potential conflict of interest.
