# Supplementary material for: Seroprevalence Surveys for Anti-SARS-CoV-2 Antibody in Different Populations in Taiwan With Low Incidence of COVID-19 in 2020 and Severe Outbreaks of SARS in 2003
Source: Front Immunol. 2021 May 18;12:626609. doi: 10.3389/fimmu.2021.626609 (PMC8167053; doi:10.3389/fimmu.2021.626609)
Supplement: Supplementary file 1 [file Table_1.docx]

**Supplementary Table:** **National Strategies for the Control of Coronavirus Disease 2019 (COVID-19) in Taiwan**

| Component | Main containment strategies | Representative population | Epidemiological implication for seroprevalence in the indicated study population |
| --- | --- | --- | --- |
| Border control | 1. Symptom screening and fever detection on landing 2. Obligated quarantine or isolation at home or designated hotel 3. Travel restrictions | Returned travelers or foreigners for international business | Influx pressure of SARS-CoV-2 |
| Healthcare system response | 1. Entrance patient diversion and streaming 2. ED outdoor space extension 3. Epidemic outpatient clinic 4. Designated quarantine ward 5. Enhanced traffic control bundle 6. Patient flow-centered infection control | Healthcare workers, especially those who take care of confirmed COVID-19 patients | Effectiveness of implementation of hospital COVID-19 infection prevention measures |
| Public engagement | 1. Wearing mask 2. Hand hygiene 3. Social distancing 4. Avoid gathering 5. Encouraging vaccination^a^ | Citizens without specific exposure risk | Previous outbreak severity  Effectiveness of community epidemic prevention action  Population vulnerability  Herd immunity establishment |

^a^COVID-19 vaccines were not available at the time of conducting this study.
